# The Correlation of Serum Uric Acid Levels With the Severity of Coronary Artery Disease in Diabetic Patients: A Cross-Sectional Study

**DOI:** 10.7759/cureus.50755

**Published:** 2023-12-18

**Authors:** Sher W Khan, Ayesha Fayyaz, Ikram Ullah, Maryam Shahab, Kainath Naeem, Bilal Ahmad, Sayeeda M Shah

**Affiliations:** 1 Cardiology, Lady Reading Hospital MTI (Medical Teaching Institution), Peshawar, PAK; 2 Cardiology, State University of New York Downstate Medical Center, New York, USA; 3 Internal Medicine, Rheumatology and Allergy Institute of Connecticut, LLC, Manchester, USA; 4 Obstetrics and Gynecology, Khyber Teaching Hospital, Peshawar, PAK

**Keywords:** cad severity, coronary artery angiogram, diabetes mellitus, coronary artery disease, serum uric acid level

## Abstract

Background and aim

Coronary artery disease (CAD) is a severe and life-threatening complication in patients with diabetes, resulting in significant morbidity and death burden globally. Although serum uric acid levels have been linked to the aetiology of both CAD and diabetes, the association between uric acid and CAD severity in diabetic patients remains unknown. This study aimed to investigate the relationship between serum uric acid levels and the severity of CAD in patients with diabetes undergoing coronary angiography. This study also compared patient parameters and comorbidities linked with high uric acid levels.

Material and methods

This cross-sectional study was conducted at the Lady Reading Hospital in Peshawar, Pakistan, from October 20, 2022, to September 20, 2023. A total of 290 patients with diabetes were enrolled. These participants were divided into groups depending on their serum uric acid levels: Group A (n = 145) and Group B (n = 145). On average, patients in Group A had high serum uric acid levels, whereas those in Group B had normal serum uric acid levels. Coronary angiograms were analysed using well-established assessment methods to determine the severity of CAD using the Syntax score as the mean score was greater for Group A with higher serum uric acid levels than Group B.

Results

The mean age of patients in Group A was 59.2±7.1 years, whereas in Group B, it was 60.5±6.8 years. The percentage of male patients in Group A was 62% and 58.6% in Group B. The mean BMI for group A was 28.4±2.3 kg/m^2^, while the mean BMI for group B was 27.9±2.1 kg/m^2^. In both groups, the prevalence of hypertension, dyslipidemia and family history of CAD did not differ significantly. Group A's mean serum uric acid levels were 8.17 ± 1.64, while in Group B, 5.03 ± 1.09. Similarly, the mean Syntax score, which is a visual estimate of CAD burden and complexity, was higher in Group A (37.59 ± 3.41) compared to Group B (26.44 ± 2.97), and the difference was statistically significant (p = 0.001). The severity of CAD based on syntax score was found to be significantly different in both groups (p=0.04).

Conclusion

This study illustrates that patients with high uric acid levels are more likely to have CAD as indicated by a higher mean Syntax score in Group A compared to Group B. However, serum uric acid levels alone cannot accurately predict the severity of CAD on coronary angiography in diabetic patients. These findings add to the evidence already available, emphasizing the significance of serum uric acid as a potential biomarker for risk stratification in this vulnerable population.

## Introduction

Coronary artery disease (CAD) is still one of the leading causes of morbidity and death worldwide, and those with diabetes have a significantly increased risk of developing CAD [1]. The inflammatory atherosclerotic condition known as CAD can cause stable or unstable angina, myocardial infarction (MI), or unexpected cardiac death. Age, gender, income level, and other factors are associated with CAD. Numerous strongly correlated processes, including lipid abnormalities, thrombosis, inflammation, remodelling, platelet activation, vascular smooth cell activation, oxidative stress, altered matrix metabolism, and hereditary variables, are associated with atherosclerosis. Multiple members of the general public have risk factors for CAD such as hypertension, diabetes mellitus, chronic renal disease, lipid and lipoprotein metabolic abnormalities, age, gender, lifestyle, cigarette smoking, nutrition, obesity, and family history [2]. Diabetes has a complicated relationship with CAD as diabetes not only increases susceptibility to CAD but also frequently leads to more severe coronary artery involvement [3]. In this context, one growing topic of study is the significance of serum uric acid levels, which have been linked to the aetiology of both CAD and diabetes [4].

The byproduct of human purine metabolism, uric acid, not only causes gout but may also contribute to the development of chronic renal disease, heart failure, hypertension, atrial fibrillation, CAD, and cardiovascular mortality. Serum uric acid has been shown to be a prognostic measure for cardiovascular outcomes in a number of clinical studies [5,6]. Hyperuricemia, or elevated serum uric acid levels, has been linked to several kinds of metabolic diseases, including hypertension, insulin resistance, and dyslipidemia, all of which are established risk factors for CAD [7,8]. Furthermore, hyperuricemia has been associated with endothelial dysfunction and oxidative stress, which are important factors in the development and progression of atherosclerosis [9,10,11]. 

Despite these findings, the link between serum uric acid levels and the severity of CAD in diabetic patients is still not entirely known, especially in our setup. Understanding this link is critical because it might lead to better risk classification, early intervention, and personalized treatment methods for this high-risk group. Therefore, this study was conducted to ascertain the relationship of serum uric acid levels with the severity of CAD in diabetic patients undergoing coronary angiography.

## Materials and methods

Objectives and setting

The study's main objective was to assess the relationship between serum uric acid levels and the severity of CAD in diabetic patients undergoing angiography. The study was conducted at Lady Reading Hospital Peshawar, Pakistan, from October 20, 2022, to September 20, 2023. Lady Reading Hospital Peshawar's Institutional Review Board (IRB) approved the study with approval number 948/LRH/MTI. All research participants provided informed consent.

Participant selection criteria

The inclusion criteria were: adults aged 18 years or older, both males and females with diabetes, who were referred from coronary angiography. On the other hand, patients with acute renal damage or stage 4 or higher chronic kidney disease, gout, or a history of using uric acid-lowering medications were excluded. This was because their medication affect the uric acid levels, which can affect study results, as thiazide diuretics cause hyperuricemia. Patients who had an MI within the last four weeks and women who were pregnant or breastfeeding were also excluded.

Sampling and data analysis

In this study, 290 diabetic patients were enrolled based on their medical history and were divided into two groups depending on their serum uric acid levels: Group A (n=145) with high serum uric acid levels and Group B (n=145) with normal serum uric acid levels. The cut-off value set for high uric acid levels was 7.0 mg/dL in the case of males and 6.0 mg/dL in the case of females. For all participants, baseline demographic and clinical data were obtained, including age, gender, BMI, smoking history, duration of diabetes, and medication use (e.g., anti-diabetic medicine, anti-hypertensive drugs). Each participant had blood samples drawn, and serum uric acid levels were determined using standardized laboratory procedures. To determine the severity of CAD, all subjects underwent coronary angiography. Competent interventional cardiologists reviewed the angiograms. The severity of CAD was determined using the Syntax score. This grading system considers coronary artery lesions' number, location, and severity. Syntax score from 16-22 was labelled as low, 22-32 was intermediate, and above 33 it was scored high. 

IBM SPSS Statistics for Windows, Version 22.0 (Released 2013; IBM Corp., Armonk, New York, Unitd States) was used to analyze the data, and a p-value of 0.05 was considered statistically significant. Means (standard deviation) or percentages are used to represent descriptive statistics for baseline attributes. For continuous variables, student's t-tests were used. The chi-square test was used to compare categorical variables. Pearson's or Spearman's correlation coefficients were employed to analyze the relationship between serum uric acid levels and CAD severity.

## Results

As per the gender distribution, 62% of Group A and 58.6% of Group B were male. The mean age of patients in Group A was 59.2±7.1 years, whereas the mean age in Group B was 60.5±6.8 years. The mean BMI for Group A was 28.4±2.3kg/m^2^, while the mean BMI for Group B was 27.9±2.1kg/m^2^. As per the BMI ranges, the most prevalent range in both groups was 30-34.9 kg/m^2^. The majority of patients in both groups, 69% in Group A and 72.4% in Group B, were nonsmokers. The proportion of past and present smokers in both groups was equal, indicating a balanced smoking history among the research participants. Diabetes duration trends were comparable in both groups. Patients with diabetes for 5-10 years were the most prevalent in both groups, accounting for 37.9% in Group A and 34.5% in Group B. Diabetes lasted an average of 8.3±3.2 years in Group A and 8.1±3.0 years in Group B. There were no statistically significant variations in gender, age, or smoking history. Both groups had a balanced gender ratio and a similar age distribution, indicating that these parameters were adequately controlled (as shown in Table 1).

**Table 1:** Characteristics of Group A and Group B (N=290) SD: standard deviation: BMI: body mass index Data given as n (%) unless otherwise mentioned.

Characteristic	Group A, High Uric Acid (n = 145)	Group B, Low Uric Acid (n = 145)	P value
Gender			
- Male	90 (62%)	85 (58.6%)	0.548
- Female	55 (38%)	60 (41.4%)	
Age (years)			
- Mean± SD	59.2±7.1	60.5±6.8	
- <50	30 (20.7%)	35 (24.1%)	0.594
- 50-60	60 (41.4%)	55 (37.9%)	
- 60-70	40 (27.6%)	45 (31.0%)	
- >70	15 (10.3%)	10 (6.9%)	
BMI (kg/m²)			
- Mean± SD	28.4± 2.3	27.9±2.1	
<25	18 (12.4%)	22 (15.2%)	0.733
- 25-29.9	45 (31.0%)	50 (34.5%)	
- 30-34.9	60 (41.4%)	55 (37.9%)	
- ≥35	22 (15.2%)	18 (12.4%)	
Smoking History			
- Non-Smokers	100 (69%)	105 (72.4%)	0.749
- Former Smokers	30 (21%)	25 (17.2%)	
- Current Smokers	15 (10%)	15 (10.3%)	
Duration of Diabetes (years)			
- Mean ±SD	8.3±3.2	8.1± 3.0	
- <5 years	35 (24.1%)	38 (26.2%)	0.807
- 5-10 years	55 (37.9%)	50 (34.5%)	
- 10-15 years	30 (20.7%)	27 (18.6%)	
- >15 years	25 (17.2%)	30 (20.7%)	

As shown in Table 2, in Group A, 20.7% of patients were prescribed insulin, whereas 19.3% used insulin. Oral hypoglycemic medicines were used to treat most patients in both groups, with 79.3% in Group A and 80.7% in Group B utilizing these drugs. Angiotensin-converting enzyme (ACE) inhibitors were used by 27.6% of Group A patients and 26.2% of Group B patients. Beta-blockers were administered to 17.2% of Group A patients and 17.9% of Group B patients. Similarly, 15.2% of Group A and 13.8% of Group B took calcium channel blockers, while 12.4% of Group A and 13.1% of Group B used diuretics. In addition, patients in both groups were using various anti-hypertensive drugs, accounting for 11.7% of Group A and 12.4% of Group B. The distribution of anti-diabetic and anti-hypertensive drugs across the two groups is generally equal and there is no significant difference observed in both groups. 

**Table 2:** Use of medications in both groups ACE: angiotensin-converting enzyme

Medications	Group A, High Uric Acid (n=145)	Group B, Low Uric Acid (n=145)	P-value
Anti-Diabetic Medications (n)			0.442
- Insulin	30 (20.7%)	28 (19.3%)	
- Oral Hypoglycemic Agents	115 (79.3%)	117 (80.7%)	
Anti-Hypertensive Drugs (n)			0.368
- ACE Inhibitors	40 (27.6%)	38 (26.2%)	
- Beta-blockers	25 (17.2%)	26 (17.9%)	
- Calcium Channel Blockers	22 (15.2%)	20 (13.8%)	
- Diuretics	18 (12.4%)	19 (13.1%)	
- Other (Specify)	17 (11.7%)	18 (12.4%)	

Mean serum uric acid levels in Group A were 8.17 ± 1.64, while in Group B, it was 5.03 ± 1.09 (Table 3). Of patients in group A, 10.3% had mild CAD, 37.9% had moderate CAD, and 51.7% had severe CAD. In Group B, 17.2% of the patients had mild CAD, 44.8% had moderate CAD, and 37.9% had severe CAD. So a significant difference in the distribution of CAD severity in both groups (p=0.040) was noticed based on serum uric acid levels.

**Table 3:** Serum uric acid and severity of coronary artery disease measurement SD: standard deviation; CAD: coronary artery disease Data is given as n (%) and mean±SD

Serum Uric Acid Levels (mg/dL)	Group A, High Uric Acid (n=145)	Group B, Low Uric Acid (n=145)	P-value
Means ± SD	8.17 ± 1.64	5.03 ± 1.09	0.001
Severity of Coronary Artery Disease (CAD) based on syntax score			
Mean Syntax score	37.59 ± 3.41	26.44 ± 2.97	0.001
- Mild CAD (0-22)	15 (10.3%)	25 (17.2%)	0.040
- Moderate CAD (23-32)	55 (37.9%)	65 (44.8%)	
- Severe CAD (≥33)	75 (51.7%)	55 (37.9%)	

Table 4 demonstrated the multiple logistic regression results where the dependent variable was taken as Syntax score, the covariates were age, gender, and serum uric acid level. In this case, a positive significant association was found between high Syntax score and uric acid levels.

**Table 4:** Comparison of Syntax score with serum uric acid levels ^a^ The reference category is low

syntax_range^a^		B		Std. Error	Wald	Sig.	Exp (B)	95% CI for Exp (B)	
								Lower Bound	Upper Bound
Intermediate	Intercept	5.612		1.952	8.266	.004			
	Uric acid	.003		.169	.000	.984	1.003	.721	1.396
	Age	-.044		.024	3.280	.070	.957	.912	1.004
	Gender	-1.669		.614	7.381	.007	.188	.057	.628
High	Intercept	1.675		1.760	.907	.341			
	Uric acid	.724		.158	20.933	.000	2.063	1.513	2.813
	Age	-.029		.022	1.806	.179	.971	.930	1.014
	Gender	-1.458		.523	7.780	.005	.233	.084	.648

Table 5 indicates the distribution of common risk variables among the research participants. In Group A, 75.9% of the patients had hypertension. With normal uric acid levels, Group B showed a somewhat reduced prevalence of hypertension, with 72.4% of patients being hypertensive. Of patients in Group A, 86.2% had dyslipidemia, whereas 82.8% in Group B had dyslipidemia. The distribution of dyslipidemia was comparable across the two groups, with both having a high prevalence of this risk factor. In group A, 51.7% of patients had a family history of CAD, whereas 48.3% did not. In Group B, 44.8% had a CAD family history, whereas 55.2% did not. This indicates that the study successfully matched the groups based on these shared risk factors and there was no significant difference between both groups based on risk factors.

**Table 5:** Risk factors of both groups. Data presented as n (%)

Risk Factors	Group A, High Uric Acid (n=145)	Group B, Low Uric Acid (n=145)	p-value
Hypertension (n)			0.686
- Yes	110 (75.9%)	105 (72.4%)	
- No	35 (24.1%)	40 (27.6%)	
Dyslipidemia (n)			0.258
- Yes	125 (86.2%)	120 (82.8%)	
- No	20 (13.8%)	25 (17.2%)	
Family History of CAD (n)			0.241
- Yes	75 (51.7%)	65 (44.8%)	
- No	70 (48.3%)	80 (55.2%)	

## Discussion

The findings of this study shed light on the link between serum uric acid levels and the severity of CAD in diabetic patients who had coronary angiography. The study found a statistically significant association between serum uric acid levels and the severity of CAD in this high-risk cohort. Group A, which had higher serum uric acid levels, had considerably more burden of CAD than Group B, as indicated by a high mean Syntax score. Even after controlling for possible confounders, including age, gender, and other cardiovascular risk factors, there was a statistically significant association.

In previous studies by Rahmani et al. and Johnson et al., the authors noted a positive association between serum uric acid levels and the severity of CAD [12,13], similar to our study which also demonstrated a positive association. However, new research revealed that uric acid levels were a less accurate predictor of CAD severity [14]. 

The findings of the current study align with other studies about the frequency of risk factors among individuals with diabetes and CAD. A study conducted by Al-Daghri et al. revealed that 72.5% of individuals diagnosed with diabetes also had hypertension [15], a finding that aligns with the prevalence rate of 75.9% seen in group A of our research. Similarly, research conducted by Kuwabara et al. revealed a dyslipidemia prevalence of 81.5% among individuals with diabetes [16], a figure that aligns closely with the 86.2% seen in group A of the current study.

Furthermore, the current study effectively ensured comparability between groups by matching them based on these shared risk variables. A greater occurrence of hyperuricemia in patients with diabetes and CAD was observed in our study in comparison to other studies. In research conducted by Wani et al. [17], the prevalence of hyperuricemia in diabetes patients was 35.5%. In the present study, we observed a prevalence of 46.9% in group A and 41.4% in group B. The observed discrepancy may be attributed to differences in the characteristics of the study sample and the research methods used. Our investigation primarily targeted patients diagnosed with diabetes and CAD, while Al-Mumin's study included all CAD patients, irrespective of their diabetes status. Furthermore, the current study used a more extensive sample size and a more precise characterisation of hyperuricemia, defined as serum uric acid levels of 6.0 mg/dL in females and 7.0 mg/dL in males, in contrast to the study conducted by Al-Mumin, which defined hyperuricemia as serum uric acid levels surpassing 7.0 mg/dL for both genders [18].

The present study will be a valuable addition to the existing literature on this important topic. It will provide a platform for further research on the association of serum uric acid levels with the severity of CAD.

Limitations

This study has some limitations. Firstly, it is a single-centre study. Secondly, the study has a cross-sectional design. Both of these could limit its generalizability; therefore, prospectively designed multicenter studies should be conducted on this topic.

## Conclusions

This cross-sectional study illustrates that diabetic patients with hyperuricemia are more likely to have CAD, as indicated by a high mean Syntax score in patients with high uric acid levels compared to those with low serum uric acid levels. However, serum uric acid levels alone cannot predict the severity of CAD in these patients. Therefore, other factors like comorbid conditions along with serum uric acid should also be considered for risk stratification of such patients.
